# Validation of T2- and diffusion-weighted magnetic resonance imaging for mapping intra-prostatic tumour prior to focal boost dose-escalation using intensity-modulated radiotherapy (IMRT)

**DOI:** 10.1016/j.radonc.2019.07.030

**Published:** 2019-12

**Authors:** E.J. Alexander, J.R. Murray, V.A. Morgan, S.L. Giles, S.F. Riches, S. Hazell, K. Thomas, S.A. Sohaib, A. Thompson, A. Gao, D.P. Dearnaley, N.M. DeSouza

**Affiliations:** a The Royal Marsden NHS Foundation Trust, Sutton, UK; b The Institute of Cancer Research, Sutton, UK

**Keywords:** TTMPB, transperineal template-guided mapping prostate biopsies, DIL, dominant intra-prostatic tumour lesion, WM-RP, whole-mount radical prostatectomy, MCCL, maximum cancer core length, DMBZ, delineatemodified Barzell zones, IPL, intraprostatic tumour lesion, Prostate cancer, Image-guided radiotherapy, Prostate radiotherapy, Magnetic resonance imaging, Mapping biopsies, Diagnostic accuracy

## Abstract

**Background and purpose:**

To assess the diagnostic accuracy and inter-observer agreement of T2-weighted (T2W) and diffusion-weighted (DW) magnetic resonance imaging (MRI) for mapping intra-prostatic tumour lesions (IPLs) for the purpose of focal dose-escalation in prostate cancer radiotherapy.

**Materials and methods:**

Twenty-six men selected for radical treatment with radiotherapy were recruited prospectively and underwent pre-treatment T2W+DW-MRI and 5 mm spaced transperineal template-guided mapping prostate biopsies (TTMPB). A ‘traffic-light’ system was used to score both data sets. Radiologically suspicious lesions measuring ≥0.5 cm^3^ were classified as red; suspicious lesions 0.2–0.5 cm^3^ or larger lesions equivocal for tumour were classified as amber. The histopathology assessment combined pathological grade and tumour length on biopsy (red = ≥4 mm primary Gleason grade 4/5 or ≥6 mm primary Gleason grade 3). Two radiologists assessed the MRI data and inter-observer agreement was measured with Cohens’ Kappa co-efficient.

**Results:**

Twenty-five of 26 men had red image-defined IPLs by both readers, 24 had red pathology-defined lesions. There was a good correlation between lesions ≥0.5 cm^3^ classified “red” on imaging and “red” histopathology in biopsies (Reader 1: *r* = 0.61, *p* < 0.0001, Reader 2: *r* = 0.44, *p* = 0.03). Diagnostic accuracy for both readers for red image-defined lesions was sensitivity 85–86%, specificity 93–98%, positive predictive value (PPV) 79–92% and negative predictive value (NPV) 96%. Inter-observer agreement was good (Cohen’s Kappa 0.61).

**Conclusions:**

MRI is accurate for mapping clinically significant prostate cancer; diffusion-restricted lesions ≥0.5 cm^3^ can be confidently identified for radiation dose boosting.

## Highlights

• 5 mm mapping prostate biopsies correlated with imaged intra-prostatic tumour.
• Diffusion-restricted tumour of ≥0.5 cm^3^ can be dose-escalated with confidence.
• Tumours of <0.5 cm^3^ should not be dose-escalated.
• Diffusion-weighted MR has good diagnostic accuracy for dominant tumour lesions.

Although dose-escalation to the whole prostate gland improves biochemical control of prostate cancer, it is at the expense of increased rectal toxicity [1], [2], [3], [4], [5], [6], [7]. The most important site for local recurrence is the dominant intra-prostatic tumour lesion (DIL) [8], [9], [10], [11] suggesting that focal radiation boosts to the DIL may improve the therapeutic ratio of prostate radiotherapy [12], [13]. Therefore, to achieve this improvement, the accuracy of imaging to detect the DIL needs to be established.

Diffusion-weighted MR is the most widely used multi-parametric magnetic resonance imaging (mpMR) parameter for detecting and staging prostate cancer, because its quantitation is correlated with Gleason grade [14], [15], [16], [17], [18], [19], [20]. Much of the histopathological correlation between mpMRI and histopathology has used the gold standard of whole-mount radical prostatectomy specimens (WM-RP), restricting analysis of imaging to patients suitable for radical prostatectomy. There is little data correlating mpMR with whole-gland histology in patients treated with radiotherapy, and in particular to define DIL’s which might be suitable for radiation boosts.

Mathematical modelling suggests transperineal template mapping prostate biopsies (TTMPB) with 5 mm spacing detects lesions ≥0.125 cc with 95% certainty [21]. Clinical studies have shown the accuracy to detect a cancer volume of 0.2 cc or greater and 0.5 cc or greater is in order of 90–95% respectively with 5 mm sampling [22], [23]. Five mm sampling (giving a sampling density of approximately 1 core per millilitre) have been shown to have a 95% sensitivity and negative predictive value (NPV) for detecting clinically significant prostate cancer (defined as ≥0.5 cm^3^ or Gleason ≥7), although this is significantly reduced with 1 cm mapping [24], [25]. If clinically significant lesions are defined as ≥0.5 cm^3^ then 5 mm TTMPB detects 96–100% of such lesions [21], [22]. A study classifying tumour burden from TTMPB core biopsy samples found that a single core with maximum cancer core length (MCCL) of 6 mm or greater had sensitivity to detect more than 95% of lesions of 0.5 cm^3^ (approximating to a 1 cm diameter lesion). A 4 mm MCCL detected more than 95% of 0.2 cm^3^ lesions [23]. The present study was designed to assess the diagnostic accuracy and inter-observer agreement of T2W+DW-MRI for mapping IPLs, using TTMPB as the reference-standard, for the purpose of focal dose-escalation in patients selected for prostate cancer radiotherapy. This is the key first step in defining DIL for boost therapy as tested in Phase 3 trials such as FLAME (NCT01168479) and PIVOTALBoost (ISRCTN80146950).

## Materials and methods

### Study design and patient population

This single institution prospective study was a sub-group of the DELINEATE trial (ISRCTN04483921). Consenting patients were recruited sequentially. The trial was approved by the local institutional review board and Regional Ethics Committee and performed in accordance with European Union guidelines for Good Clinical Practice. Hormone-naïve patients with National Comprehensive Cancer Network (NCCN) [26] intermediate or high risk localised prostate cancer were eligible, patients with seminal vesicle involvement were excluded. All patients had standard staging investigations prior to recruitment. Eight weeks after the diagnostic trans-rectal ultrasound-guided biopsies, patients underwent an MRI comprising of T2W and DW-MR followed by a TTMPB procedure.

### MR acquisition

MR imaging was performed on a 1.5T whole-body MR scanner (Avanto, Siemens, Erlangen). Data were acquired using an endorectal receiver coil (ERC) inflated with 60mls of air in combination with an external phased array body coil. A 20 mg intramuscular injection of butylscopolamine bromide (Buscopan, Boehringer Ingelheim) was administered to reduce peristalsis. The MR protocol comprised slice-matched, 3-mm, transverse T2W fast spin-echo and single-shot echo-planar DWI MRI to cover the entire prostate gland. T2W fast spin-echo images were also acquired in sagittal and coronal planes. ADC maps were generated from all b values 0–800 s/mm^2^ (parameter details in Supplementary Appendix A).

### TTMPB procedure

Patients were anaesthetised, given prophylactic antibiotics and set-up in the lithotomy position. Biopsies were taken at 5 mm intervals, apical and basal aspects of the prostate were biopsied separately if prostate length required. Cores were taken by an experienced urologist, blinded to the MR results, and each core was marked with ink at the apical end to define polarity [27]. The supra-urethra area was avoided to prevent urethral injury.

### Imaging and histopathology interpretation

**Fig. 1:**
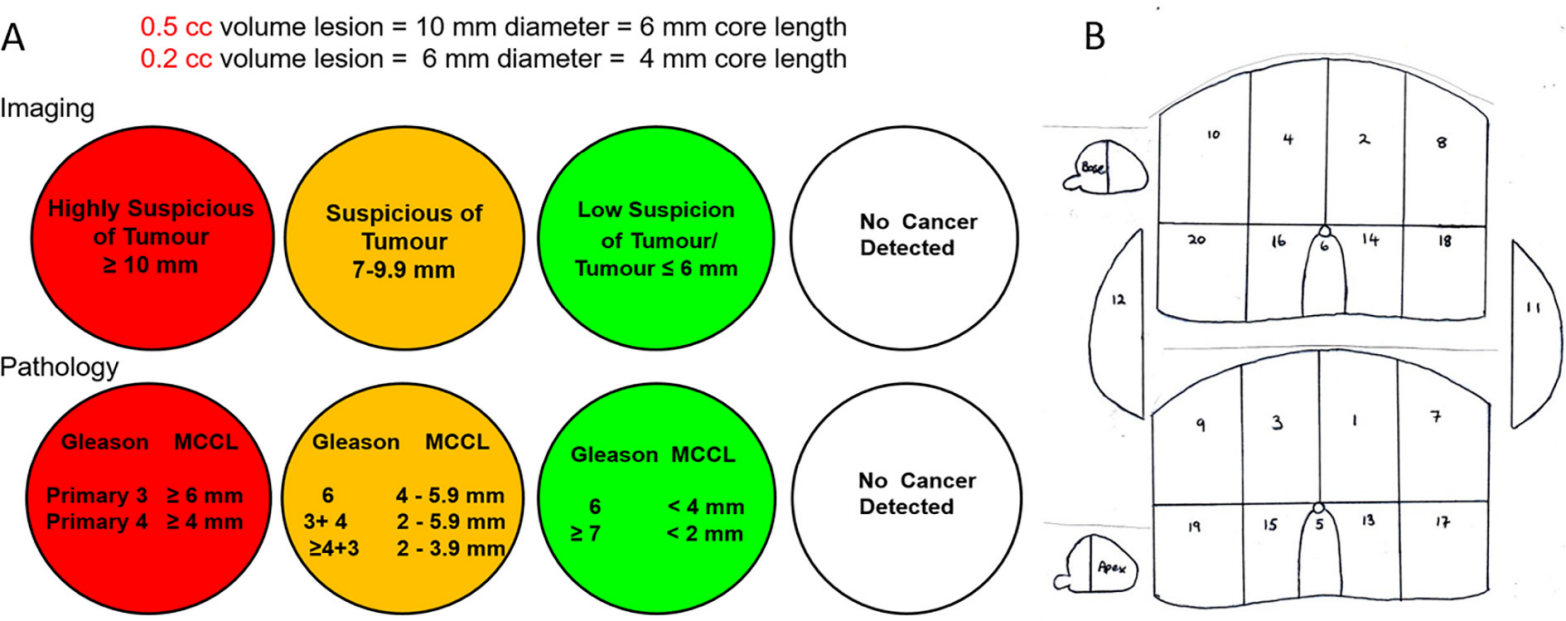
Schematic representation of (A) imaging and pathological traffic light classification and (B) delineate-modified Barzell zones (see Supplemental Appendix B).

**Fig. 2:**
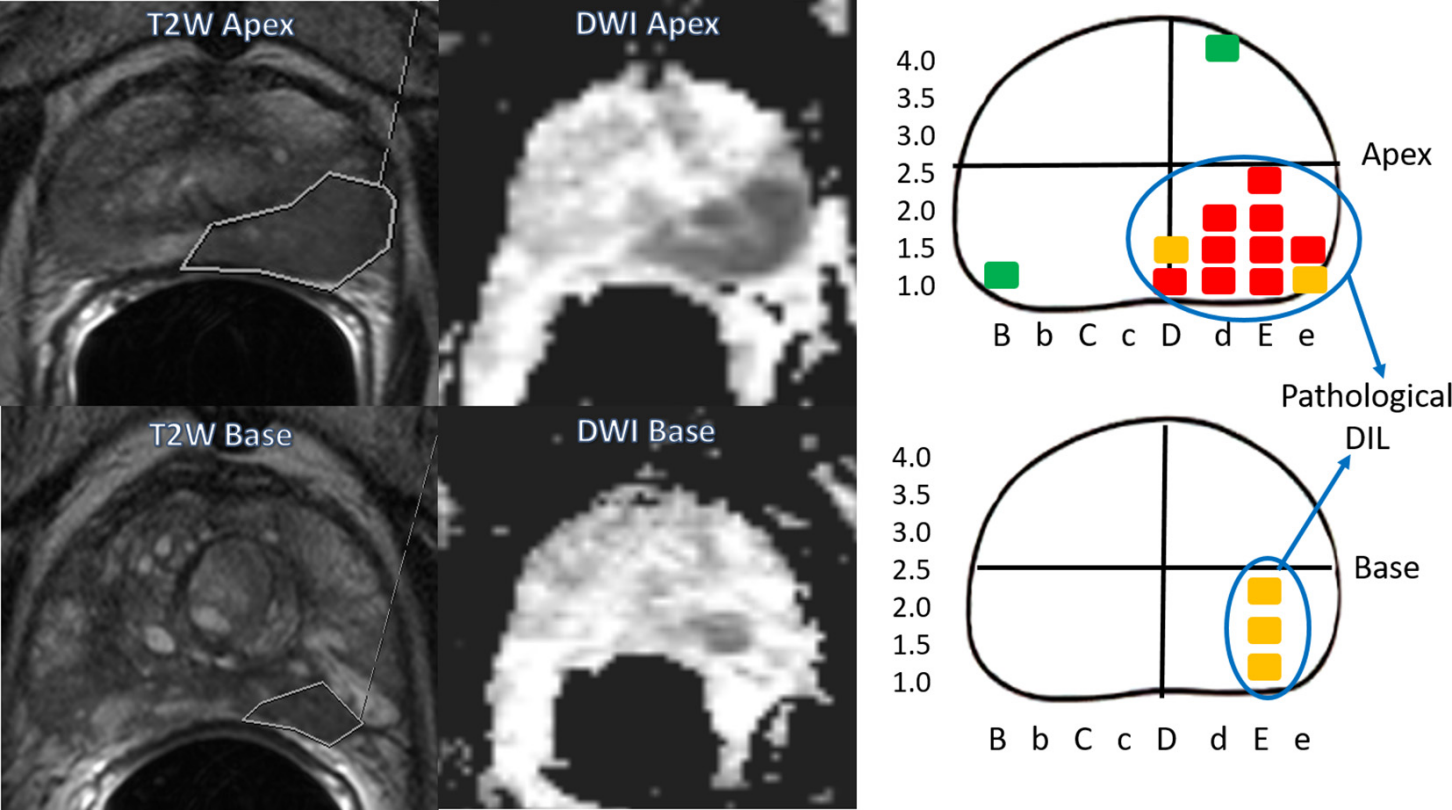
T2W and DWI MR images of patient 16 taken at apical and basal segments of the prostate gland and compared to schematic diagram plotting positive biopsies in the patient’s transperineal template-guided prostate mapping biopsies, showing the cores that would be considered part of the pathological DIL.

Two uro-radiologists qualitatively and independently read the T2W+DW-MRI data. Both radiologists were blinded to the clinical patient data. Tumour was defined as a low signal-intensity focal lesion on T2W that showed restricted diffusion on DW-MRI. In the transitional zone, the homogeneity of the lesion and its mass effect was also considered in order to differentiate it from stromal nodules. The prostate was analysed in octants and in Barzell zones [28], which were modified for analysis taking into account the size of the prostate gland. If the prostate was short in length the analysis was performed as a single layer making quadrants or 11 Delineate-modified Barzell zones (DMBZ) (Fig. 1B and Supplemental Appendix B). Each sector was classified with a traffic-light system as red, amber, green or white. Red corresponded to tumour ≥10 mm in diameter (≥0.5 cm^3^) on imaging or MCCL ≥6 mm of primary Gleason grade 3 or ≥4 mm primary Gleason grade ≥4 on TTMPB. Amber corresponded to tumour between 7–9.9 mm (0.2–0.49 cm^3^)/abnormality equivocal for tumour on imaging or MCCL 4–6 mm of primary Gleason grade 3 or ≥2 mm of ≥primary Gleason grade ≥4. Green corresponded to tumour ≤6 mm diameter (≤0.2 cm^3^) or low suspicion of tumour on imaging or MCCL <4 mm of Gleason 6 or <2 mm of Gleason ≥7. White corresponded to no tumour on imaging or biopsy (Fig. 1A). Each sector was analysed using 2 thresholds; true positive if imaging and pathology sectors were classified as (1) both red or (2) both either red or amber. Green and white were not considered to be clinically significant prostate cancer lesions. If less than 1/3 on a sector on biopsy was affected by tumour it was classified as negative. The DMBZ analysis was performed with both strict and flexible methods. The flexible method allowed for minor geographical mismatch between sectors; if an imaged sector was positive where corresponding pathology sector was negative, any directly adjacent positive pathology zones were classified as a true positive [29]. The DIL was defined as the largest lesion identified by both readers. The second IPL was defined as the next largest lesion identified by one or both readers. The pathological DIL and other IPLs were defined considering the total cancer core length contained in clustered positive biopsies i.e. all adjacent/contiguous biopsies (Fig. 2).

### Statistical analysis

Statistical analysis was performed in Excel (Microsoft) and Software Package for Social Sciences (SPSS® v21.0, IBM Corp, NY, USA) following a pre-specified Statistical Analysis Plan (Supplemental Appendix B). Descriptive statistics were used to assess tumour volumes and diameters. Sensitivity, specificity, positive predictive value (PPV) and negative predictive value (NPV) by sector with binomial 95% confidence intervals (CI) were calculated with Wald adjustments. All sectors were combined to give diagnostic accuracy measurements for each reader. ROC curves compared the AUC for each reader for significant cancer detection. Inter-observer agreement was measured with Cohen’s Kappa coefficient [30] and interpreted as: 0–0.2 slight agreement, 0.21–0.4 fair agreement, 0.41–0.6 moderate agreement, 0.61–0.8 good agreement, ≥0.81 almost perfect agreement. Spearman rank correlation assessed relationships between imaging tumour volumes and pathological findings. The pre-specified primary end-point was the diagnostic performance using DMBZ with the red only, flexible methodology, other endpoints were regarded as exploratory.

## Results

Twenty-six eligible and consenting patients were recruited between October 2010 and November 2013 (56 patients were recruited to the whole study’s initial phase). Patient characteristics are shown in Table 1. Seventeen patients (65%) had intermediate risk and 9 (35%) high risk disease. The median interval between diagnostic trans-rectal ultrasound-guided biopsies and study MR was 12.2 weeks.

**Table 1:** Summary of patient characteristics (IQR = interquartile range).

Patient characteristics	N = 26
Median age (IQR)	70.5 (66–74)

Clinical stage/radiological stage	
T1c	9/0
T2 a/b	11/12
T2 c	1/9
T3a	5/5

Gleason Score at diagnosis	
3 + 3	5 (19%)
3 + 4	14 (54%)
4 + 3	6 (23%)
≥8	1 (4%)

PSA	
Median (IQR)	9.5 (5.6–17.25)

NCCN Risk Classification [1]	
Intermediate	17 (65%)
High	9 (35%)

Interval between diagnostic trans-rectal ultrasound-guided biopsies and study MR (Days)	
Median (IQR)	85 (69–184)

Interval between study MR and transperineal template-guided prostate mapping biopsies (Days)	
Median (IQR)	16.5 (11–25)
Prostate volume	39.5 (32–55)
No of cores taken	45 (38–56)
Sampling Density (cores/cc)	1.1 (0.9–1.3)

Median prostate volume was 39.5 cm^3^ (IQR 32–55 cm^3^) with a median sampling density of 1.1 core/cm^3^ (IQR 0.9–1.3 core/cm^3^). Prostate carcinoma was found in all patients. Red biopsies were found in 24 out of 26 patients (92%); 421 DMBZ were analysed (median 20 [IQR 11–20] per patient respectively). Traffic-light classification was; 66/421 (15.7%) of DMBZ were red and 30/421 (7.1%) of DMBZ were amber. Twelve patients (46%) had no prostate carcinoma sampled outside the imaged IPLs. The remainder of patients (54%) had positive biopsies outside imaged IPLs; 1 patient had a single amber core (3 mm Gleason 3 + 4), 13 patients had green cores (0.5–3 mm Gleason 3 + 3). No patient had red biopsy cores outside the imaged IPLs.

**Fig. 3:**
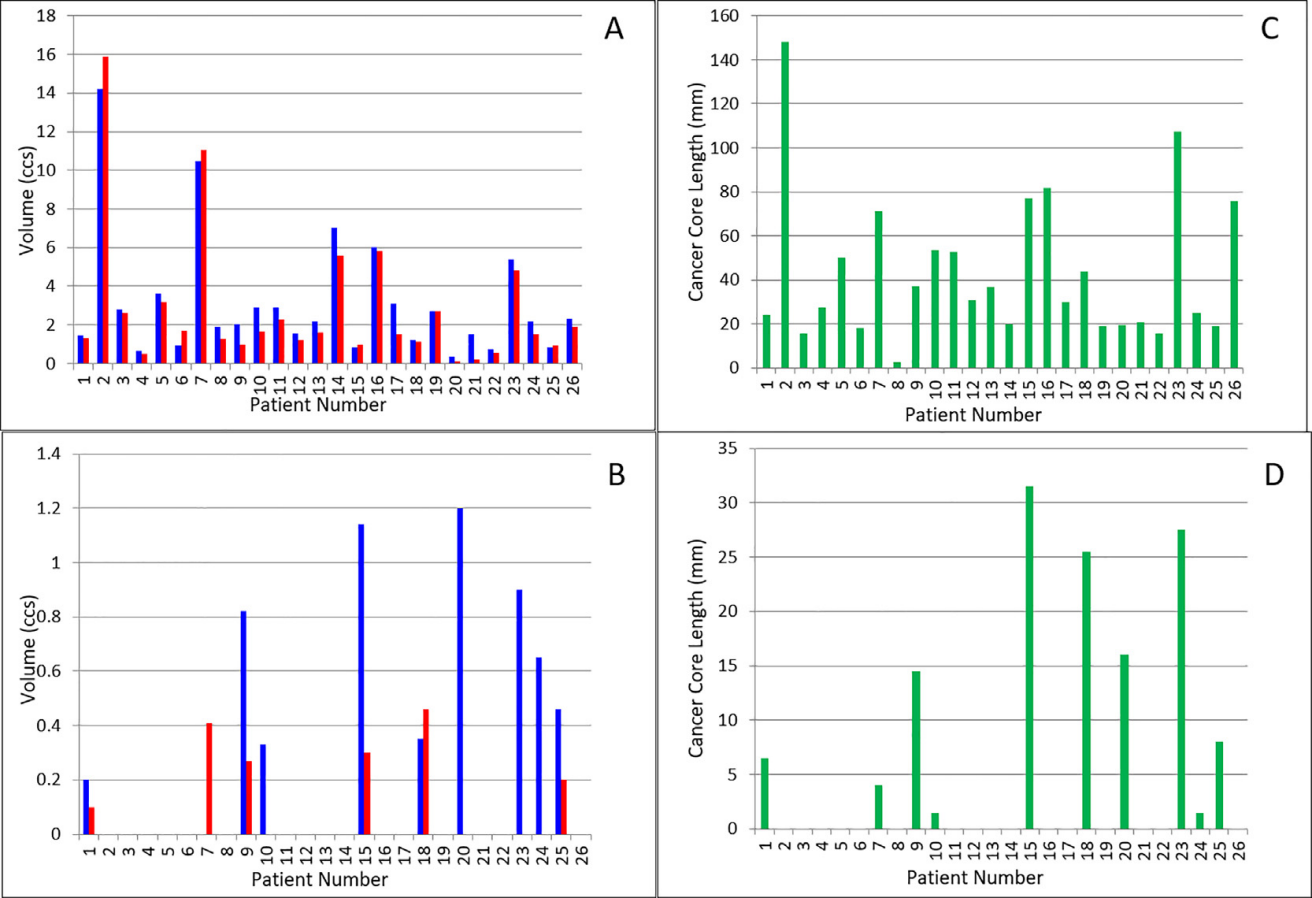
Measured volumes for Reader 1 (Blue) and Reader 2 (Red) of (A) dominant intraprostatic lesion, (B) second largest intraprostatic lesion, (C) cancer core length for DIL and (D) cancer core length for 2nd IPL. Note differing scales for each figure.

The diagnostic accuracy parameters for the T2W+DW-MR images for readers 1 and 2 are shown in Table 2. The diagnostic accuracy of T2W+DW-MRI was high for both readers for identification of tumour within a given sector of the prostate; sensitivity 85–86%, specificity 93–98%, PPV 79–92% and NPV 96%. Cohen’s kappa statistic for inter-observer agreement was 0.61 indicating good agreement between the 2 readers. Median DIL volumes (Fig. 3A) were 2.2 cm^3^ (IQR 1.4–3.1 cm^3^) for reader 1 and 1.54 cm^3^ (IQR 1.1–2.7 cm^3^) for reader 2. A 2nd IPL was recorded on MR in 11 patients by reader 1 (median volume 0.63 cm^3^ (IQR 0.34–0.88 cm^3^) and in 5 patients by reader 2 (median volume 0.29 cm^3^ (IQR 0.27–0.38 cm^3^) (Fig. 3A + B).

**Table 2:** Diagnostic accuracy parameters for T2W+DWI for both readers for Delineate modified Barzell zones (DMBZ) flexible method for both pathological thresholds (PPV = Positive Predictive value, NPV = Negative Predictive Value, AUC = Area under the ROC curve).

Pathological Threshold		Prevalence %	Sensitivity % (95%CI)	Specificity % (95%CI)	PPV % (95%CI)	NPV % (95%CI)		AUC
DMBZ flexible red only imaging path threshold red	Reader 1	23	85 (77–91)	93 (90–96)	79 (70–86)	96 (93–97)		0.87 (0.83–0.92)
	Reader 2	22	86 (78–92)	98 (96–99)	92 (84–96)	96 (93–98)		0.94 (0.91–0.98)

DMBZ flexible red and amber imaging path threshold red and amber	Reader 1	30	80 (72–86)	94 (91–96)	86 (78–91)	92 (88–94)		0.88 (0.84–0.93)
	Reader 2	28	78 (69–84)	98 (95–99)	93 (86–97)	92 (88–94)		0.92 (0.89–0.95)

Cancer core lengths in DILs and 2nd IPLs are shown in Fig. 3C + D. There was a statistically significant correlation between both the imaged DIL volume and any imaged red lesion volume with total cancer core length in that volume; reader 1: *r* = 0.44 and 0.61, *p* = 0.026 and 0.0001 respectively; reader 2: *r* = 0.50 and 0.44, *p* = 0.01 and 0.03 respectively (Table 3). Correlation between the 2nd IPL volume or any imaged amber lesion volume was poorer and did not reach statistical significance. Exploratory endpoints including amber as well as red categories, assessment using octants and strict rather than flexible DMBZ definitions and are shown in Supplementary Appendix C. The various methods showed sensitivity 67–87%, specificity 84–98%, PPV 49–91% and NPV 89–96%.

**Table 3:** Spearman correlation coefficients for Total Cancer Core Lengths and MR defined lesions by readers 1 and 2.

	Spearman correlation (*ρ*) between MR volume and pathological criteria (P value)			
	Total cancer core length in DIL	Total cancer core length in 2nd IPL	Total cancer core length in imaged-defined red lesions	Total cancer core length in image-defined amber lesions
Reader 1	0.44 (*p* = 0.026)	0.66 9 (*p* = 0.03)	0.61 (*p* < 0.0001)	0.73 (*p* = 0.88)

Reader 2	0.5 (*p* < 0.01)	0.57 (*p* = 0.18)	0.44 (*p* = 0.03)	0.32 (*p* = 0.478)

## Discussion

DELINEATE is the first prospective study to our knowledge assessing the diagnostic accuracy of MR for the purposes of defining the position of a radiation boost in a population planned for radical treatment with radiotherapy rather than prostatectomy. TTMPB at 5 mm intervals were used as the reference standard to achieve whole gland pathological sampling.

This study has shown that T2W+DW-MRI has good diagnostic accuracy for mapping the location and extent of tumour lesions measuring ≥0.5 cm^3^ or ≥1 cm diameter with restricted diffusion on MR. Sensitivity, specificity and NPV was consistently high by 2 independent observers for the primary outcome measure; red only, flexible method (85–86%, 93–98% and 96% respectively), although the PPV varied more significantly (79–92%) due to the low prevalence of total sectors affected by tumour. The inclusion of amber lesions in the analysis caused a small decrease in sensitivity (78–80%) and NPV (92%). This was due to a marginal decrease in false positives with a corresponding larger increase in false negatives.

Other small, single institution series have referenced MR with WM-RP but vary in technical parameters used and so are not strictly comparable [17], [31], [32], [33], [34]. Reported sensitivities and specificities vary widely from 23 to 96% [35], [36], [37] mainly reflecting the prevalence of the disease, the reason for performing the test, the variability in the threshold level for defining positive disease and the sector level selected for analysis, all of which critically influence the reported results.

Two complementary studies have recently reported assessments of MR in the diagnostic pathway for prostate cancer. The UK PROMIS paired-cohort multicentre trial [38] investigated mpMRI (T2W, DWI and dynamic contrast-enhanced (DCE) MR) to define primary Gleason 4 or cancer of any grade ≥6 mm (0.5 cm^3^) on TTMPB, demonstrating a sensitivity, specificity, PPV and NPV of 93%, 41%, 51% and 89% respectively. However, as the primary end-point was cancer diagnosis, the threshold chosen for a positive MR was lower and considered the whole prostate as a single entity. When the PROMIS data is assessed with only lesions likely to represent cancer (exclusion of equivocal lesions), sensitivity, specificity, PPV and NPV are 70%, 78%, 70% and 84% respectively, which remain lower than but more comparable with our work. This may be explained by the different population of patients examined, improved imaging resolution using an endorectal coil and the multicentre nature of the PROMIS data. A similar prospective multicentre Australian study of 344 men assessed T2W/DWI/DCE using the PI-RADS scale [19] in 344 men with a cut-off of equivocal for positive MR. [39]. Significant prostate cancer was defined as Gleason 7 with more than 5% grade 4, ≥20% of cores positive or ≥7 mm of prostate cancer in any core on transperineal template-guided prostate biopsies (median 30 cores per patient). Sensitivity, specificity, PPV and NPV of 96%, 36%, 52% and 92% were reported respectively which are very similar to PROMIS. Anatomical concordance of the location of imaged lesion and significant cancer on biopsy was found in 97%. The lack of 5 mm mapping is likely to have impacted on the ability to detect all clinically significant cancer as the size of tumour left undetected is directly related to the uniform spacing between core samples [21]. The consistent results of the PROMIS and Australian multicentre studies results suggest that our findings will be generalisable.

For initial MR screening it is desirable to keep the false negative rate as low as possible. For focal dose-escalation, where the remainder of the prostate is getting standard doses of radiation, it is preferable to keep the false positive rates as low as possible, i.e., a higher specificity. Dose-escalation to false positives could cause increased toxicity without additional benefit, thereby reducing any improvement in therapeutic ratio. In our data only 2 patients (8%) had a false positive MR when assessing the whole prostate; one had multiple adjacent amber cores (multiple cores of 2–5.5 mm Gleason grade 4 + 3). The second had multiple adjacent cores with <4 mm of tumour in the basal sections of the cores, classified as green, suggesting inadequate sampling of the imaged basal tumour.

Inter-observer agreement between the readers and correlation between the delineated MR volumes (for the DIL and red lesions) and the total cancer core length was good, largely a consequence of the size of the DIL and the higher Gleason grade of these tumours causing substantial diffusion-restriction on the DW-MRI. Correspondingly, smaller lesions of lower Gleason grade were more difficult to define and led to poor agreement between radiologists and poorer correlation with total cancer core length. The inclusion of these lesions for dose boosting is questionable both because of the imaging uncertainty and lack of need to boost smaller cancer foci. Reassuringly, inter-observer agreement in the multi-centre PROMIS trial (0.63 Cohen’s Kappa) was similar to that in our study. In future, a combination of T2W and diffusion-weighted imaging will generate contrast for more accurate and even semi-automated GTV delineation.

There are several limitations to our study. First, the number of patients was small. Second, despite the extensive sampling some areas of prostate are difficult to fully biopsy without undue risks to patients. These areas include the extreme base of the gland (bladder neck injury), the supra-urethral area (urethral injury) and pubic arch interference limiting access to the most anterior part of larger prostate glands. In our patients this caused “false positives” to be scored on a minority of imaging sectors. Third, prostate biopsies may underestimate the true tumour burden by sampling the periphery rather than the centre of smaller lesions. Although this risk is reduced with 5 mm mapping, it will have had an effect on the analysis of total cancer core length as a surrogate for pathological volume. Fourth, we acknowledge the statistical analysis assesses all DMBZ and octants as independent of each other within each patient. This is however a well-documented approach to sector-based diagnostic accuracy studies [29], [40], [41], [42]. Finally, we acknowledge that there is inevitably some uncertainty related to the mapping of the prostate images to the stylised Barzell diagram which certainly will have introduced minor geographical discrepancies between the reporting radiologists and the pathological assessments.

We have shown that T2W+DW-MRI robustly identifies DIL for focal boost radiotherapy, the accuracy of which underpins clinical evaluation of such approaches. The DELINEATE trial has now recruited over 200 patients using conventional or modest hypofractionation schedules. A recent systematic review identified 988 patients treated with a DIL radiation boost within Phase1/2 studies which appear to be associated with low toxicity [43] even with prolonged follow-up of 8 years [44]. DIL boosts are being assessed in ongoing clinical phase 3 trials such as FLAME (NCT01168479) [45], [46] and PIVOTALboost (ISRCTN80146950).

In summary, focal dose escalation to DIL may be limited to lesions ≥1 cm in diameter (≥0.5 cm^3^), where T2W+DW-MRI imaging suggest a high suspicion of tumour which can be defined with confidence. Lesions <0.5 cm^3^ or larger lesions less restricted on DW-MRI should be treated with standard radiation doses. Including these lesions in the threshold for focal boosts increases false positives and risks increasing toxicity without therapeutic benefit.

## References

[1] Dearnaley D.P., Sydes M.R., Graham J.D., Aird E.G., Bottomley D., Cowan R.A. (2007). Escalated-dose versus standard-dose conformal radiotherapy in prostate cancer: first results from the MRC RT01 randomised controlled trial. Lancet Oncol.

[2] Zietman A.L., DeSilvio M.L., Slater J.D., Rossi C.J., Miller D.W., Adams J.A. (2005). Comparison of conventional-dose vs high-dose conformal radiation therapy in clinically localized adenocarcinoma of the prostate: a randomized controlled trial. JAMA.

[3] Al-Mamgani A., van Putten W.L., Heemsbergen W.D., van Leenders G.J., Slot A., Dielwart M.F. (2008). Update of Dutch multicenter dose-escalation trial of radiotherapy for localized prostate cancer. Int J Radiat Oncol Biol Phys.

[4] Beckendorf V., Guerif S., Le Prise E., Cosset J.M., Bougnoux A., Chauvet B. (2011). 70 Gy versus 80 Gy in localized prostate cancer: 5-year results of GETUG 06 randomized trial. Int J Radiat Oncol Biol Phys.

[5] Pollack A., Zagars G.K., Starkschall G., Antolak J.A., Lee J.J., Huang E. (2002). Prostate cancer radiation dose response: results of the M. D. Anderson phase III randomized trial. Int J Radiat Oncol Biol Phys.

[6] Peeters S.T., Lebesque J.V., Heemsbergen W.D., van Putten W.L., Slot A., Dielwart M.F. (2006). Localized volume effects for late rectal and anal toxicity after radiotherapy for prostate cancer. Int J Radiat Oncol Biol Phys.

[7] Kuban D., Pollack A., Huang E., Levy L., Dong L., Starkschall G. (2003). Hazards of dose escalation in prostate cancer radiotherapy. Int J Radiat Oncol Biol Phys.

[8] Arrayeh E., Westphalen A.C., Kurhanewicz J., Roach M., Jung A.J., Carroll P.R. (2012). Does local recurrence of prostate cancer after radiation therapy occur at the site of primary tumor? Results of a longitudinal MRI and MRSI study. Int J Radiat Oncol Biol Phys.

[9] Pucar D., Hricak H., Shukla-Dave A., Kuroiwa K., Drobnjak M., Eastham J. (2007). Clinically significant prostate cancer local recurrence after radiation therapy occurs at the site of primary tumor: magnetic resonance imaging and step-section pathology evidence. Int J Radiat Oncol Biol Phys.

[10] Cellini N., Morganti A.G., Mattiucci G.C., Valentini V., Leone M., Luzi S. (2002). Analysis of intraprostatic failures in patients treated with hormonal therapy and radiotherapy: implications for conformal therapy planning. Int J Radiat Oncol Biol Phys.

[11] Chopra S., Toi A., Taback N., Evans A., Haider M.A., Milosevic M. (2012). Pathological predictors for site of local recurrence after radiotherapy for prostate cancer. Int J Radiat Oncol Biol Phys.

[12] Riches S.F., Payne G.S., Desouza N.M., Dearnaley D., Morgan V.A., Morgan S.C. (2014). Effect on therapeutic ratio of planning a boosted radiotherapy dose to the dominant intraprostatic tumour lesion within the prostate based on multifunctional MR parameters. Br J Radiol.

[13] Nutting C.M., Corbishley C.M., Sanchez-Nieto B., Cosgrove V.P., Webb S., Dearnaley D.P. (2002). Potential improvements in the therapeutic ratio of prostate cancer irradiation: dose escalation of pathologically identified tumour nodules using intensity modulated radiotherapy. Br J Radiol.

[14] desouza N.M., Reinsberg S.A., Scurr E.D., Brewster J.M., Payne G.S. (2007). Magnetic resonance imaging in prostate cancer: the value of apparent diffusion coefficients for identifying malignant nodules. Br J Radiol.

[15] deSouza N.M., Riches S.F., Vanas N.J., Morgan V.A., Ashley S.A., Fisher C. (2008). Diffusion-weighted magnetic resonance imaging: a potential non-invasive marker of tumour aggressiveness in localized prostate cancer. Clin Radiol.

[16] Morgan V.A., Kyriazi S., Ashley S.E., DeSouza N.M. (2007). Evaluation of the potential of diffusion-weighted imaging in prostate cancer detection. Acta Radiol.

[17] Haider M.A., van der Kwast T.H., Tanguay J., Evans A.J., Hashmi A.T., Lockwood G. (2007). Combined T2-weighted and diffusion-weighted MRI for localization of prostate cancer. AJR Am J Roentgenol.

[18] Yoshimitsu K., Kiyoshima K., Irie H., Tajima T., Asayama Y., Hirakawa M. (2008). Usefulness of apparent diffusion coefficient map in diagnosing prostate carcinoma: correlation with stepwise histopathology. J Magn Reson Imaging.

[19] Barentsz J.O., Richenberg J., Clements R., Choyke P., Verma S., Villeirs G. (2012). ESUR prostate MR guidelines 2012. Eur Radiol.

[20] Dickinson L., Ahmed H.U., Allen C., Barentsz J.O., Carey B., Futterer J.J. (2011). Magnetic resonance imaging for the detection, localisation, and characterisation of prostate cancer: recommendations from a European consensus meeting. Eur Urol.

[21] Kepner G.R., Kepner J.V. (2010). Transperineal prostate biopsy: analysis of a uniform core sampling pattern that yields data on tumor volume limits in negative biopsies. Theor Biol Med Model.

[22] Crawford E.D., Rove K.O., Barqawi A.B., Maroni P.D., Werahera P.N., Baer C.A. (2013). Clinical-pathologic correlation between transperineal mapping biopsies of the prostate and three-dimensional reconstruction of prostatectomy specimens. Prostate.

[23] Ahmed H.U., Hu Y., Carter T., Arumainayagam N., Lecornet E., Freeman A. (2011). Characterizing clinically significant prostate cancer using template prostate mapping biopsy. J Urol.

[24] Crawford E.D., Wilson S.S., Torkko K.C., Hirano D., Stewart J.S., Brammell C. (2005). Clinical staging of prostate cancer: a computer-simulated study of transperineal prostate biopsy. BJU Int..

[25] Valerio M., Anele C., Charman S.C., van der Meulen J., Freeman A., Jameson C. (2016). Transperineal template prostate-mapping biopsies: an evaluation of different protocols in the detection of clinically significant prostate cancer. BJU Int..

[26] Mohler J, Bahnson RR, Boston B, Busby JE, D’Amico A, Eastham JA, et al. NCCN clinical practice guidelines in oncology: prostate cancer. J Natl Compr Canc Netw. 8:162–200. 10.6004/jnccn.2010.0012 20141676

[27] Onik G., Miessau M., Bostwick D.G. (2009). Three-dimensional prostate mapping biopsy has a potentially significant impact on prostate cancer management. J Clin Oncol.

[28] Barzell W.E., Melamed M.R. (2007). Appropriate patient selection in the focal treatment of prostate cancer: the role of transperineal 3-dimensional pathologic mapping of the prostate–a 4-year experience. Urology.

[29] Turkbey B., Pinto P.A., Mani H., Bernardo M., Pang Y., McKinney Y.L. (2010). Prostate cancer: value of multiparametric MR imaging at 3 T for detection-histopathologic correlation. Radiology.

[30] Cohen J. (1960). A coefficient of agreement for nominal scales. Educ Psychol Measur.

[31] Futterer J.J., Heijmink S.W., Scheenen T.W., Veltman J., Huisman H.J., Vos P. (2006). Prostate cancer localization with dynamic contrast-enhanced MR imaging and proton MR spectroscopic imaging. Radiology.

[32] Graser A., Heuck A., Sommer B., Massmann J., Scheidler J., Reiser M. (2007). Per-sextant localization and staging of prostate cancer: correlation of imaging findings with whole-mount step section histopathology. AJR Am J Roentgenol.

[33] Villeirs G.M., Oosterlinck W., Vanherreweghe E., De Meerleer G.O. (2010). A qualitative approach to combined magnetic resonance imaging and spectroscopy in the diagnosis of prostate cancer. Eur J Radiol.

[34] Scheidler J., Hricak H., Vigneron D.B., Yu K.K., Sokolov D.L., Huang L.R. (1999). Prostate cancer: localization with three-dimensional proton MR spectroscopic imaging–clinicopathologic study. Radiology.

[35] Kirkham A.P., Emberton M., Allen C. (2006). How good is MRI at detecting and characterising cancer within the prostate?. Eur Urol.

[36] de Rooij M., Hamoen E.H., Futterer J.J., Barentsz J.O., Rovers M.M. (2014). Accuracy of multiparametric MRI for prostate cancer detection: a meta-analysis. AJR Am J Roentgenol.

[37] Futterer J.J., Briganti A., De Visschere P., Emberton M., Giannarini G., Kirkham A. (2015). Clinically significant prostate cancer be detected with multiparametric magnetic resonance imaging? A systematic review of the literature. Eur Urol.

[38] Ahmed H.U., El-Shater Bosaily A., Brown L.C., Gabe R., Kaplan R., Parmar M.K. (2017). Diagnostic accuracy of multi-parametric MRI and TRUS biopsy in prostate cancer (PROMIS): a paired validating confirmatory study. The Lancet.

[39] Thompson J.E., van Leeuwen P.J., Moses D., Shnier R., Brenner P., Delprado W. (2016). The diagnostic performance of multiparametric magnetic resonance imaging to detect significant prostate. Cancer J Urol.

[40] Pucar D., Shukla-Dave A., Hricak H., Moskowitz C.S., Kuroiwa K., Olgac S. (2005). Prostate cancer: correlation of MR imaging and MR spectroscopy with pathologic findings after radiation therapy-initial experience. Radiology.

[41] Coakley F.V., Teh H.S., Qayyum A., Swanson M.G., Lu Y., Roach M. (2004). Endorectal MR imaging and MR spectroscopic imaging for locally recurrent prostate cancer after external beam radiation therapy: preliminary experience. Radiology.

[42] Sala E., Eberhardt S.C., Akin O., Moskowitz C.S., Onyebuchi C.N., Kuroiwa K. (2006). Endorectal MR imaging before salvage prostatectomy: tumor localization and staging. Radiology.

[43] von Eyben F.E., Kiljunen T., Kangasmaki A., Kairemo K., von Eyben R., Joensuu T. (2016). Radiotherapy boost for the dominant intraprostatic cancer lesion-a systematic review and meta-analysis. Clin Genitourin Cancer.

[44] Sundahl N., De Meerleer G., Villeirs G., Ost P., De Neve W., Lumen N. (2016). Combining high dose external beam radiotherapy with a simultaneous integrated boost to the dominant intraprostatic lesion: analysis of genito-urinary and rectal toxicity. Radiother Oncol.

[45] Lips I.M., van der Heide U.A., Haustermans K., van Lin E.N., Pos F., Franken S.P. (2011). Single blind randomized Phase III trial to investigate the benefit of a focal lesion ablative microboost in prostate cancer (FLAME-trial): study protocol for a randomized controlled trial. Trials.

[46] Monninkhof E.M., van Loon J.W.L., van Vulpen M., Kerkmeijer L.G.W., Pos F.J., Haustermans K. (2018). Standard whole prostate gland radiotherapy with and without lesion boost in prostate cancer: Toxicity in the FLAME randomized controlled trial. Radiother Oncol.

