# Supplementary material for: Validation of T2- and diffusion-weighted magnetic resonance imaging for mapping intra-prostatic tumour prior to focal boost dose-escalation using intensity-modulated radiotherapy (IMRT)
Source: Radiother Oncol. 2019 Dec;141:181–7. doi: 10.1016/j.radonc.2019.07.030 (PMC6908966; doi:10.1016/j.radonc.2019.07.030)
Supplement: Supplementary data 1 [file mmc1.docx]

**Supplementary Appendices A-C**

**Supplementary Appendix A**

|  | **TR (ms)** | **TE (ms)** | **FOV**  **(mm^2^)** | **Slice Thickness**  **(mm)** | **Slices** | **Matrix** | **Gap (mm)** | **NSA** | **Flip Angle** | **Other** |
| --- | --- | --- | --- | --- | --- | --- | --- | --- | --- | --- |
| **Localisers** | 7 | 2.4 | 400 | 8 | 3x3 each localiser | 256x179 | 1.6 AP  4 SI/RL | 2 | 20 |  |
| **T2W Sagittal** | 5500 | 108 | 140 | 3 | 19 | 256 x 230 | 0.3 | 2 | 142 |  |
| **T2W Axial Small FOV** | 6250 | 108 | 140 | 3 | 22 | 256 x 256 | 0 | 3 | 137 |  |
| **T2W Coronal** | 5460 | 108 | 140 | 3 | 16 | 256 x 256 | 0.3 | 2 | 144 |  |
| **T2W True axial** | 5500 | 96 | 140 | 3 |  | 256 x | 0 | 2 | 70 |  |
| **DWI** | 4200 | 101 | 190 | 3 | 22 | 128 x 128 | 0.6 | 1 | 134 | b values 0, 100, 300,500 and 800 s/mm^2^  FSS |
| **T2 map** | 3200 | 30/60/90/120 | 190 | 3 | 22 | 128 x 128 | 0 | 2 | 180 |  |
| **T1W Large FOV** | 500 | 12 | 350 | 6 | 40 | 256 x 256 | 0.6 | 1 | 134 |  |
| **T2W Large FOV** | 9620 | 84 | 350 | 6 | 40 | 320 x 240 | 0.6 | 1 | 120 |  |
|  |  | | | | | | | | | |
|  |  | | | | | | | | | |

Table 1 Multi-parametric acquisition parameters of T2W/DWI MRI protocol. FSS = fat saturation suppression

**Supplementary Appendix B Statistical Analysis Plan**

**Delineate Sub-StudyTemplate Biopsies and Imaging Correlation Statistical Analysis Plan**

**Defining the Pathological Classification for Traffic Light Scoring System**

Assessing tumour volume and true tumour burden is a limitation of any biopsy technique even with the extensive number of biopsies taken with TTMPB. Disease burden from core biopsies must be correlated with the likelihood or probability to represent a certain burden of cancer. Data have been published in this area by a group from UCLH, London, who have extensive experience of TTMPB. A computer modelling study was performed using 107 whole mount histopathology specimens from radical prostatectomy patients (WM-RRP) and 500 simulations of 5 mm mapping with TTMPB per WM-RRP patient, varying needle trajectory pathways and simulating deformations of the prostate gland [[1](#_ENREF_1)]. They used the 2 published definitions of clinically significant prostate cancer proposed by Goto [[2](#_ENREF_2)] and Epstein [[3](#_ENREF_3)], 0.5 cc and 0.2 cc respectively, to quantify the cancer core lengths in core biopsy specimens that would have a 95% sensitivity of detecting lesions of these respective sizes.

A total of 665 cancer foci were examined. The maximum cancer core length (MCCL) in a single core biopsy that detected more than 95% of lesions of 0.5 cc was 6 mm or greater, for lesions of 0.2 cc this was 4 mm or greater. In addition to the core length cut-off threshold, the group combined the Gleason score pattern in order to define 2 definitions of clinically significant cancer that can be used to classify prostatic carcinoma found on TTMPB. Clinically significant disease could be classified as below, with red and amber representing clinically significant disease and green representing clinically insignificant disease (see Figure 1). Any disease that contained Gleason score of 7 was considered clinically significant with higher weighting on disease of primary Gleason grade 4.

It is important to note that although these definitions have not as yet been validated, no similar work attempting to model or quantify core lengths with WM-RRP histology has been published to date, this therefore is the best available evidence.

Figure 1. Definitions of clinically significant (red and amber) and non-clinically significant disease (green) in localised prostate cancer[[1](#_ENREF_1)]

For the purposes of this study we wish to examine the diagnostic accuracy of magnetic resonance imaging with respect to the identification of tumour for radiation boosts. The aim of the Ahmed et al. study was the detection of clinically significant disease, which may be used to distinguish between patients that may require some form of radical treatment and those that may enter surveillance protocols. Disease which may be considered clinically significant by the UCL criteria would not necessarily require an additional radiation boost e.g. small foci (1-2 mm) of Gleason 4+3, as it would be receiving the standard dose to the remainder of the prostate gland, i.e. for the DELINEATE study protocol 74 Gy in 37 fractions. We therefore needed to extrapolate from the published data to acquire thresholds that were more reasonable for addition radiation boost rather than radical treatment per se. For the MCCL we first considered what level of disease we would want to boost. A 0.5 cc volume lesion is equivalent to a 1 cm diameter lesion. A 0.2 cc volume lesion is equivalent to a 7 mm diameter lesion. We considered any lesion a centimetre or more in diameter on imaging, would be eligible for a radiation boost. The threshold would be lowered if primary Gleason grade 4 or higher was found on template mapping biopsy where the threshold for size would be lowered to a 0.2 cc lesion due to the more aggressive histology. Secondly, we considered what grade and size of lesion we would definitely not want to boost. Those areas were considered as low grade disease of less than 0.2 cc, with lower thresholds for higher grade disease. Anything that fell between the definitions described in red and green were described as amber, areas that we were unsure that we would want to boost but would consider as possibly significant for a boost dependant on imaging results.

**Traffic Light Scoring System - Maximum Cancer Core Length**

**
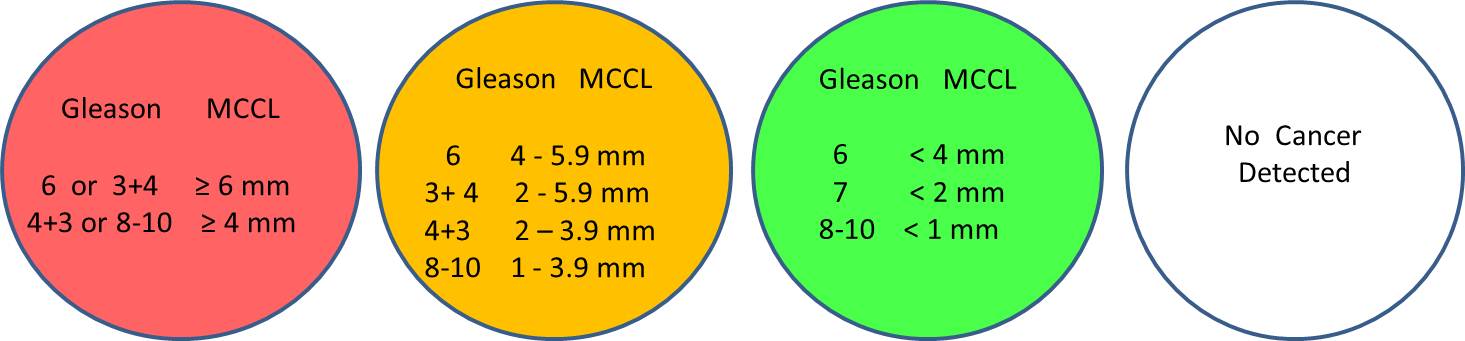
**

**Figure 2.DELINEATE Maximum Cancer Core Length Classification System. MCCL is the maximum cancer length in each individual specimen pot for that co-ordinate (being one core only, apical or basal).**

**AIM OF DELINEATE BIOPSY SUB-STUDY**

To evaluate the role of multi-parametric MRI in identifying intra-prostatic tumour for the purpose of boosting that tumour with radiation.

**Primary Objective**

- To assess the ability of mp-MRI to accurately detect tumour foci that are clinically significant enough to warrant a radiation boost

**Secondary Objectives**

- To quantify differences in accuracy with the use of T2W + DWI compared with overall mp-MRI (T2W +DWI)
- To assess inter-observer variation in imaging reporting

**Primary Outcome Measures**

1. The overall accuracy measures sensitivity, specificity, positive predictive value (PPV) and negative predictive value (NPV) (and their respective 95% confidence intervals) T2W + DWI, will be reported for the overall patient group using 2 cut-offs for positive disease, red alone (R) and red and amber (R+A).
2. The main endpoint will be to detect cancer foci as defined by the Maximum Cancer Core Length (MCCL), using both R and R+A. This will be analysed according to 2 ways of matching MR and pathology zones, firstly strict and secondly a flexible analysis which attempts to account for some of the registration errors that can occur in classifying imaging and pathology into small zones. This will account for the registration errors that can occur due to deformation of the endorectal coil and ultrasound probe and errors due to deviations of the biopsy needle track. Hence each accuracy measure will be calculated four times in total (using strict matching + red disease; strict matching + red or amber; flexible + red; flexible + red/amber). **The main primary endpoint will be MCCL, red only analysed by the flexible method.**

**Secondary Outcome Measures**

1. Inter-observer variation of readers (2 MR uro-radiologists) will be measured using the imaging datasets traffic light coding

**Exploratory Outcome Measures**

1. The accuracy measures of sensitivity and specificity T2W + DWI will be reported with the prostate divided into quadrants with each quadrant given an overall cancer score indices i.e. the DELINEATE traffic light scale(red, amber, green and white)
2. Receiver Operator Characteristic (ROC) curves will be constructed as a summary measure for each observer reading using the total patient groups’ quadrant sensitivity and 1-specificity for each cancer scoring indices (e.g. red, amber, green and white). Area under the curve (AUC) will be calculated and assessed for each observer.

**Statistical methods**

**General principles**

- Imaging and pathology regions of interest/ sectors will be defined as being independent of each other

**Primary outcome measures**

- Two definitions will be used for positive disease on pathology (positive disease is defined as clinically significant enough for a radiation boost), firstly red sectors only (R) and secondly red and amber (R+A) sectors. For all analyses green and white will be classified as negative for requiring a radiation boost. Disease will be classified using Maximum Cancer Core Length (MCCL).
- Each image will be matched to the pathology report by DELINEATE Modified Barzell Zone (DMBZ), with pathology taken as the gold standard. Two x2 tables will be constructed for each imaging modality, to report the total numbers of true and false positives and true and false negatives summed over all zones and all patients. Separate tables will be constructed for each of the two definitions of disease on pathology listed above (R or R + A).
- Analysis for octants will be performed for the strict method only.
- The sensitivity, specificity, PPV and NPV will be calculated with 95% confidence intervals

**Secondary outcome measures**

- Inter-observer variation in scores (1=Red, 2=Amber, 3=Green and White or 1 = Red and 2= Amber, Green and White) for each zone between the two MR radiologists will be compared using Cohen’s Kappa coefficient
- Similar methods to the primary analysis will be used to match imaging to pathology by quadrants rather than zones. Each quadrant will be assigned the lowest i.e. red = 1, amber= 2 or green=3 score for any zone in the quadrant. Imaging and pathology reports will then be matched and 2x2 tables showing total number of true and false negatives, true and false positives over all quadrants will be constructed. Separate tables will be constructed for each of the two definition of disease on pathology (R and R+A,)
- Receiver operative characteristic (ROC) curves with be plotted using classification of the DELINEATE traffic light score in quadrants rather than Barzell zones. For each observer, quadrants will be scored as 1(Red), 2 (Amber), 3 (Green) and 4(White). These scores will then be used to construct a ROC curve to illustrate sensitivity and specificity for each score 1-4 as a cut-off to predict for positive disease as shown on pathology. Two different ROC curves will be constructed for each observer, one for each definition of positive disease on pathology (Red using MCCL, Red + Amber using MCCL, The area under the curve (AUC) will be calculated for each definition of positive disease and each observer.
- Using similar methods as described above, two by two contingency tables will be constructed for each radiologist’s qualitative reading of the MR scans using the two definitions of positive (R alone and R+A). These tables will be constructed to show true positive, true negative, false positive and false negative for MR imaging for each radiologist using MCCL definitions.

**METHODS**

**Data Collection at time of Template Mapping Biopsy**

The patient is set-up to the correct imaging postion, this is dependant on the anatomy of the individual gland. The standard set-up is to place urethra at co-ordinate D 2.5 or 2.0. When patient is at desired set-up postion ultrasound pictures are taken to record the position of the prostatic capsule and urethra. During the biopsy procedure the samples are inked at the apical end and potted individually in order to maintain spatial resolution. A list of biopsied co-ordinates and annotations of discrepancies during the procedure is made during the procedure (e.g. whether one core only, assymetric swelling or catheter deviation, pubic arch obstruction).

**Radiology Data Collection**

Multi-parametric MRI

Two uro-radiologists experienced in MR reporting will independently qualitatively assess (visual assessment) mp-MR images and record the traffic light cancer scoring index on a CRF on a per patient basis. Zones will be classified according to the DELINEATE traffic light scale using the DELINEATE Modified Barzell Zones (DMBZ 11 - 20 zones). Individual lesions will be labelled and marked on the DMBZ on the CRF.

If 1/3 or more of a sector is positive on imaging it is classified as positive (red or amber), if less than a third of the sector is involved it should be classified as negative. Spatial resolution may mean that, in particular with the DELINEATE Modified Barzell zones, it is difficult to distinguish between such small areas. For example the lateral sectors 11 and 12, and sub-urethral zones 5 and 6, differentiation is very challenging unless the tumour is confined to the midline. These zones should therefore be considered positive unless the reader has confidence that it is not affected.

Definition of extra-pelvic nodal sites will be the bottom of L5 vertebra and above.

For each lesion marked red or amber the tumour volume will be recorded.

The definition of the midline of the gland in the AP direction is the marked by a line approximately 50% between anterior and posterior, the maximum posterior diameter should be 1.7 cm please see notes on DELINEATE Barzell zones.

**Pathological Data Collection**

The co-investigator (EA) will record the histopathology cores on the pathology CRF. The primary assessment tool for the pathology will be DMBZ. Using the cores as they have been designated (apical cores will be scored in apical Barzell zones and basal cores in basal Barzell) There will be exceptions to this as specified *a priori* here;

1. Cores that were taken as ‘one core only’ due to small size of gland – if a majority of the cores (> 80% were a single core) these data will be analysed and compared with imaging as a single depth of Barzell zones reducing the number of sectors to 11 rather than 20 zones. If this occurs the lowest scoring zone e.g. red, amber, green is counted for the imaging.
2. Cores that are ‘one core only’ because at the periphery of the gland or short cranio-caudal distance and therefore (generally) spanning the mid-gland will be placed in either the apical or basal half of the gland that correlates with positive imaging. If both apical and basal sectors are red on imaging, the core can be placed in either DMBZ, if one is amber and one is red, it may contribute to the amber zone.
3. Contiguous cancer spanning the mid-gland of the prostate may be summed together to define adjacent Barzell zones in the superior-inferior i.e. apical-basal direction, as “red” or “amber” for both pathology sectors. Contiguous cancer must be spatially in the middle of the gland (as defined by inked ends, defined as ≤ 2 mm of normal prostate tissue between stretches of tumour within adjacent apical and basal cores at the same co-ordinate). This compliments the instructions given to radiologist regarding tumour that spans the mid-gland to be judged positive for both apical and basal sectors.

**Defining a Positive Pathology sector**

- DELINEATE Modified Barzell zones (DMBZ) have predefined classification of co-ordinate into sectors (see Figure 3) based on the original Barzell zones [[4](#_ENREF_4)], but adapted to better correlate with imaging depending on gland size / width. The system shown in Figure 3 will be used. There may be exception of particularly large or small prostates e.g. large prostates with positive biopsies laterally, for example in little f, in which case column d and E will contribute towards the same zones (zones 1-4)and e and F will contribute towards the next zones (zones 7-10) with column little f only being the lateral zone 11. This is to match with the imaging reporting where zones 11 and 12 are defined as the extreme lateral side of the gland. Similarly if small prostate and little b and little e are the lateral part of the glands, these will be allocated to the lateral zones 11 and 12 and big C and big E will become single column zones
- Maximum Cancer Core Length (MCCL) per sector will be used to define whether a particular sector is classified as red, amber, green or white (see Figure 2)
- Peripheral samples that are one core only are assumed to be mid-gland unless specifically labelled as basal and those cancer cores’ length will be assigned as defined above (Path data collection - Section 1).
- The midline of the gland is defined by the urethra in both directions. Ultrasound images and notations taken at time of biopsy are used to record urethral position. The midline in the right to left direction is placed at big D unless unusual prostatic anatomy. The midline of the gland in the anterior-posterior (AP) direction is usually set at 2.5 but may be at 2.0 for a small gland, there are individual variations on occasion. This means that the posterior half of the gland will be defined as approximately the posterior 1.7 cm (or sometimes 1.2 cm in a small gland with urethra at 2.0) depending on the size of the gland and position of urethra. On occasions the prostate was unable to be lined up sufficiently well at ultrasound the urethra may be at 3.0 but no samples taken at 1.0 or 1.5. In this situation the posterior 2-3 rows of the biopsies will be defined as the posterior half of the gland, dependant on gland size.
- Positive biopsies taken along the midline of the gland in an anterior-posterior dimension i.e. the midline row, e.g. row 2.5 can contribute to either adjacent sector (or midline of gland in RL direction, i.e. big D column, for the quadrant analysis). If there is a sector defined as positive on imaging adjacent to this midline the cancer core length will be added to that pathology sector. If both adjacent sectors are “positive” or “negative” on imaging the cancer core length will contribute to either one of these zones but not both.
- For the strict method analysis strict boundary definitions will be adhered to in classification of the pathology cores into sectors except where the core lies on a boundary line e.g. midline row (usually row 2.0 or 2.5). These midline positives cores they will be designated as stated above
- For the flexible method any zone which is positive will allow the zone next to it either in the x, y or z direction to be allowed to be positive if the imaging is positive in this zone. **Diagonal zones will not be allowed to correlate positively in the flexible method**. If this is judged to be due to geographical miss, i.e. only one sector red or amber (no other adjacent zones positive) mismatched with a sector on imaging with no other adjacent zones positive it may be defined as geographical miss and the sector may be defined as a true positive with an adjacent true negative. If there are 2 neighbouring sectors positive on imaging and one zone on pathology the adjacent zone can be defined as positive on path under the flexible method and both zones will be true positives. Imaging zones will not be altered, expect in the case of geographical miss as defined in this paragraph.


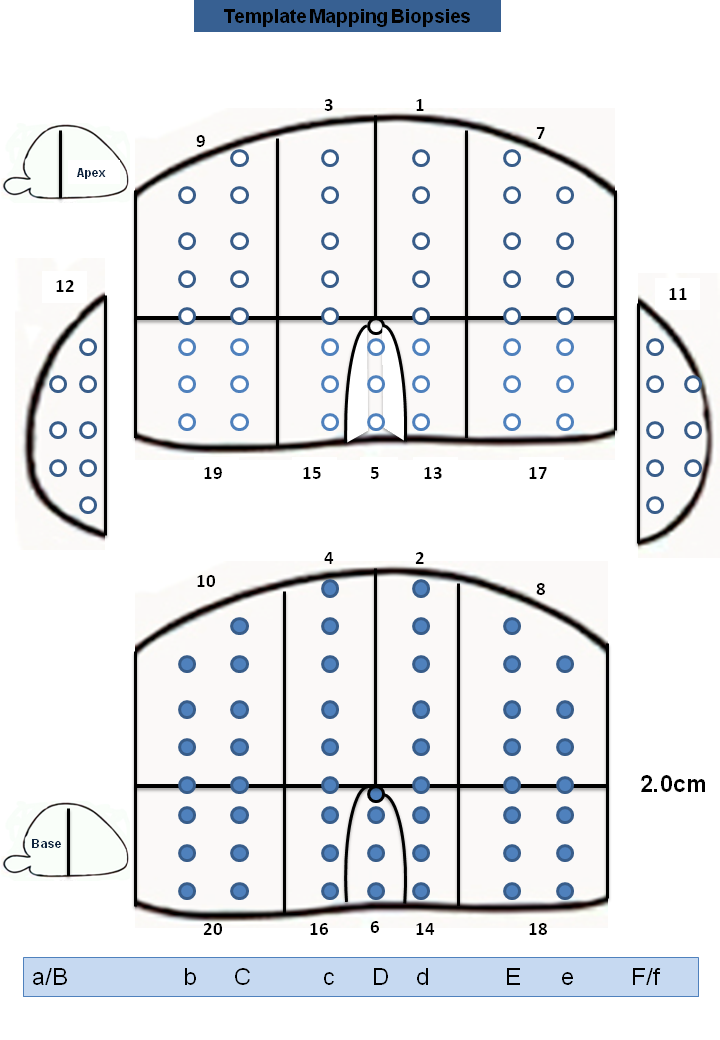


Features of Delineate Modified Barzell Zones (DMBZ)

- Medial zones 1-4 and 13-16 are 5 - 7.5 mm width (depending on whether sub or supra-urethral) containing 1 column
- Extreme lateral part of gland is the most lateral column width (5 mm maximum)
- The remaining zones 7,8,9,10 and 17,18,19,20 will be 1 cm wide (2 columns wide)
- The midline is marked by the urethra in the centre of the prostate, at 2.0 or 2.5; this generally means the posterior part of the gland is 1.2 –1.7 cm wide however ERC compression may reduce this width
- Zones 5 and 6 are sub urethral midline zones, biopsies are not taken above these in midline due to urethral injury risk

Exceptions to this occur with small or large prostate where

- Zones 7,8,9,10 and 17,18,19,20 may be 5 mm (1 column) wide (small prostate)
- Medial zones 1-4 and 13-16 may be 1 – 1.25 cm width containing 2 columns (large prostate)

Figure 3. Delineate Modified Barzell Zones with example of ‘typical’ co-ordinate columns and row set-up, each small circle represents a core biopsy from one co-ordinate

**Imaging Traffic Light System**

**Defining a Positive Imaging Sector**

If 1/3 or more of a sector is positive on imaging it is classified as positive (red or amber), if less than a third of the sector is involved it should be classified as negative.

The base and apex are divided at the point of 50% of the cranial caudal measurement of the prostate.

If a lesion spans middle of gland report both apical and basal imaging sectors are reported as positive. If abuts the mid-gland but doesn’t cross it may be defined as just apical or basal.

**Imaging Traffic Light Scoring System**


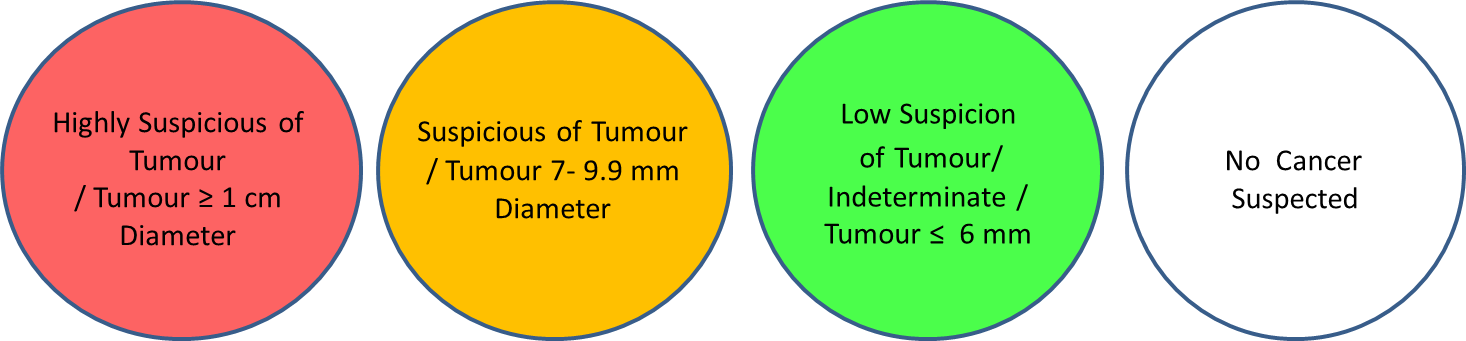


**Figure 4.Imaging DELINEATE Traffic Light Classification System**

**Defining a Positive Imaging Sector**

If 1/3 or more of a sector is positive on imaging it is classified as positive (red or amber), if less than a third of the sector is involved it should be classified as negative.

The base and apex are divided at the point of 50% of the cranial caudal measurement of the prostate.

The anterior and posterior are divided as a line approximately at point of 50% of the gland

If a lesion spans middle of gland report both apical and basal imaging sectors are reported as positive. If abuts the mid-gland but doesn’t cross it may be defined as just apical or basal.

|  | T2W | DWI |
| --- | --- | --- |
| RED | ≥ 1 cm diameter  (equivalent to 0.5 cc or >) | Homogeneous focal restricted diffusion with hyperintensity on high b value images (b800 values)without evidence of haemorrhage **over 1 cm** diameter |
| AMBER | 7 – 9.9 mm diameter (equivalent 0.2-0.49 cc) | Homogeneous focal restricted diffusion with hyperintensity on high b value images (b800 values)without evidence of haemorrhage between **7 -9.9 mm** diameter  OR  Larger lesion (≥ 1cm) with homogeneous restricted diffusion but restricted to a lesser magnitude |
| GREEN | ≤ 6 mm diameter  (<0.2 cc)  Or Indeterminate | Homogeneous focal restricted diffusion with hyperintensity on high b value images (b800 values) without evidence of haemorrhage  **≤ 6 mm** diameter  OR  Indeterminate lesions |
| WHITE | Normal prostatic tissue and benign prostatic pathology | Heterogeneous focal restricted diffusion |

**Table 1. Parameter criteria for classification within the traffic light system for MR imaging**

**Further *a-priori* stipulations**

There are situations where we anticipate that the reference standard i.e. TMPB may under-sample the prostate, due to technical limitations or self-imposed restrictions due to risk of increased complications and therefore give false positives for the imaging that are not necessarily false.

Such situations are;

1. The false positive areas are contiguous with the intra-prostatic lesion (IPL)
2. The false positive is in the basal part of the gland close to the inferior bladder
3. The false positive is adjacent to periphery of the gland, particularly anteriorly
4. The false positive is in the midline supra-urethral area

Analysis will also be performed excluding the lesions which are listed above, where the reference standard is known to have limitations in full mapping. The proportion of FPs due to these lesions will also be reported.

**References**

1. Ahmed, H.U., et al., *Characterizing clinically significant prostate cancer using template prostate mapping biopsy.* J Urol, 2011. **186**(2): p. 458-64.

2. Goto, Y., et al., *Distinguishing clinically important from unimportant prostate cancers before treatment: value of systematic biopsies.* J Urol, 1996. **156**(3): p. 1059-63.

3. Epstein, J.I., et al., *Pathologic and clinical findings to predict tumor extent of nonpalpable (stage T1c) prostate cancer.* Jama, 1994. **271**(5): p. 368-74.

4. Barzell, W.E. and M.R. Melamed, *Appropriate patient selection in the focal treatment of prostate cancer: the role of transperineal 3-dimensional pathologic mapping of the prostate--a 4-year experience.* Urology, 2007. **70**(6 Suppl): p. 27-35.

**Supplementary Appendix C Other Results**

| **Pathological Threshold** |  | **Prevalence %** | **Sensitivity % (95%CI)** | **Specificity % (95%CI)** | **PPV % (95%CI)** | **NPV % (95%CI)** | **AUC** |
| --- | --- | --- | --- | --- | --- | --- | --- |
| **DMBZ Flexible Red Only Imaging**  **Path threshold Red** | Reader 1 | 23 | 85  (77-91) | 93  (90-96) | 79  (70-86) | 96  (93-97) | 0.87  (0.83-0.92) |
|  | Reader 2 | 22 | 86  (78-92) | 98  (96-99) | 92  (84-96) | 96  (93-98) | 0.94  (0.91-0.98) |
| **DMBZ Flexible Red and Amber Imaging**  **Path threshold Red and Amber** | Reader 1 | 30 | 80 (72-86) | 94 (91-96) | 86 (78-91) | 92 (88-94) | 0.88  (0.84-0.93) |
|  | Reader 2 | 28 | 78 (69-84) | 98 (95-99) | 93 (86-97) | 92 (88-94) | 0.92  (0.89-0.95) |
| **DMBZ Strict Red Only Imaging**  **Path threshold Red** | Reader 1 | 16 | 79  (67-87) | 85  (81-88) | 49  (40-59) | 96  (93-97) | 0.80  (0.73-0.87) |
|  | Reader 2 | 16 | 76  (64-85) | 90  (86-93) | 58  (48-68) | 95  (92-97) | 0.83  (0.76-0.89) |
| **DMBZ Strict Red and Amber Imaging**  **Path threshold Red and Amber** | Reader 1 | 23 | 72 (62-80) | 84 (80-84) | 57 (48-66) | 91 (87-94) | 0.78 (0.72-.0.84) |
|  | Reader 2 | 23 | 67 (57-76) | 91 (87-93) | 68 (58-68) | 90 (86-93) | 0.78 (0.72-.0.85) |
| **Octants Red Only Imaging**  **Path threshold Red** | Reader 1 | 28 | 87  (75-94) | 85  (77-90) | 71  (58-81) | 94  (88-97) | 0.82  (0.75-0.9) |
|  | Reader 2 | 28 | 85  (74-94) | 92  (82-94) | 81  (62-85) | 94  (89-97) | 0.89  (0.82-0.96) |
| **Octants Red and Amber Imaging**  **Path threshold Red and Amber** | Reader 1 | 36 | 83  (71-91) | 84  (76-91) | 75  (63-84) | 90  (82-95) | 0.83  (0.76-0.91) |
|  | Reader 2 | 36 | 80  (68-88) | 95  (85-95) | 91  (79-98) | 89  (82-94) | 0.83  (0.76-0.91) |

Table 1.Diagnostic accuracy parameters for T2W+DWI for both readers for both pathological threshold at octant and DMBZ sector level of analysis (PPV = Positive Predictive value, NPV = Negative Predictive Value, AUC = Area under the ROC
